# Unconscious learning of likes and dislikes is persistent, resilient, and reconsolidates

**DOI:** 10.3389/fpsyg.2014.01051

**Published:** 2014-10-06

**Authors:** Alex Pine, Avi Mendelsohn, Yadin Dudai

**Affiliations:** ^1^Department of Neurobiology, Weizmann Institute of ScienceRehovot, Israel; ^2^Department of Psychiatry and Neuroscience, Friedman Brain Institute, Icahn School of Medicine at Mount SinaiNew York, NY, USA

**Keywords:** preferences, conditioning, learning, subliminal, decision-making, reward, liking, reconsolidation

## Abstract

Preferences profoundly influence decision-making and are often acquired through experience, yet it is unclear what role conscious awareness plays in the formation and persistence of long-term preferences and to what extent they can be altered by new experiences. We paired visually masked cues with monetary gains or losses during a decision-making task. Despite being unaware of the cues, subjects were influenced by their predictive values over successive trials of the task, and also revealed a strong preference for the appetitive over the aversive cues in supraliminal choices made days after learning. Moreover, the preferences were resistant to an intervening procedure designed to abolish them by a change in reinforcement contingencies, revealing a surprising resilience once formed. Despite their power however, the preferences were abolished when this procedure took place shortly after reactivating the memories, indicating that the underlying affective associations undergo reconsolidation. These findings highlight the importance of initial experiences in the formation of long-lasting preferences even in the absence of consciousness, while suggesting a way to overcome them in spite of their resiliency.

## Introduction

Humans and animals can learn to predict future reinforcement and make appropriate responses based upon knowledge of its contingency with environmental cues and actions. Experimental analysis has demonstrated that in associative learning paradigms contingent CS-US (conditioned stimulus—unconditioned stimulus) pairings (observational or via instrumental responses) have the potential to create multiple associative representations in the brain (Mackintosh, 1983; Cardinal et al., 2002; Dickinson and Balleine, 2002). Some of these associations enable stimuli to become imbued with the affective and motivational properties of the reinforcers they predict, and go on to independently influence intentional action and goal-directed behavior in a number of powerful ways (Cardinal et al., 2002; Dickinson and Balleine, 2002; Everitt et al., 2003; Berridge, 2004; Everitt and Robbins, 2005; Berridge and Aldridge, 2009).

A common manifestation of this phenomenon is that conditioning can engender a change in the hedonic evaluation of stimuli, leading to the formation of preferences (likes and dislikes), which profoundly guide behavior and choice (Rozin et al., 1998; De Houwer et al., 2001; Baeyens et al., 2005b; Hofmann et al., 2010). Indeed, it is thought that most preferences are learned rather than innate. However, the neuropsychological basis and the behavioral characteristics of these hedonic evaluations are unclear, since it is not certain whether their expression simply reflects declarative knowledge/memory of stimulus (-action)-reinforcement contingencies/pairings. A convincing account of this learning necessitates the elimination of the declarative component, but whether preferences can be acquired by humans, without conscious awareness and persist over time is currently unknown (Field, 2000; De Houwer et al., 2001; Lovibond and Shanks, 2002; Baeyens et al., 2005a; Hofmann et al., 2010). Here, we address this question in order to delineate the learning and memory characteristics of preferences, to understand their fate in response to the passage of time and new experiences, and to assess how these qualities differ from other forms of conditioned responses.

A unique feature of preferences is that they remain relatively stable over one's lifetime. This resilience has also been observed experimentally, where supraliminally acquired preferences appear to be resistant to extinction training protocols (Baeyens et al., 1988, 2005a,b; De Houwer et al., 2001; Vansteenwegen et al., 2006; Dwyer et al., 2009; Hofmann et al., 2010), though not always in all aspects (Delamater, 2007). However, such a result might be unremarkable if we assume that preferences based upon declarative memory are less affected by extinction training than are more implicitly acquired associations, in particular due to the ability to recall and re-experience initial experiences using episodic memory. Thus, by assessing whether preferences can be acquired associatively in the absence of awareness we were also able to address this issue by determining what effect a change in reward/punishment contingencies has on the degree of liking or disliking of a stimulus. If associatively learned preferences are indeed resilient to subsequent experiences and new learning, we speculated that it may be possible to harness the phenomenon of reconsolidation—a putative retrieval induced memory lability (Nader, 2003; Dudai, 2006)—to alter or abolish them. Such a finding could suggest a possible route to treatment for disorders associated with intense liking or disliking of stimuli, such as addiction and phobias. For example, might the preference for contexts (conditioned place preference) and cues associated with drug taking be amenable to a disruption of reconsolidation? Indeed, a nascent proposal (Miller and Marshall, 2005; Debiec and LeDoux, 2006; Kindt et al., 2009; Milton and Everitt, 2010; Schiller et al., 2010; Xue et al., 2012) to manipulate reconsolidation, in order to abolish the aberrant emotional salience of cues which dominate behavior in post-traumatic stress disorder and addiction, is gaining traction. But while evidence from human fear conditioning indicates that fear memories—gaged by skin conductance responses (SCRs)—undergo reconsolidation in humans (see Schiller and Phelps, 2011), it is not yet known whether a similar propensity is shown by affective properties of CSs that influence higher order behaviors such as preference formation, which are critical in the abovementioned disorders; nor whether reconsolidation in humans is specific to primary aversive conditioning or extends to appetitive and more abstract, or secondary reinforcement, as recent evidence from studies of cigarette craving would suggest (Xue et al., 2012).

To address these questions, we examined subliminal instrumental learning using appetitive and aversive secondary reinforcement in humans. Our first aim was to determine if instrumental behaviors and preferences to discriminatory stimuli can be acquired without conscious awareness, and if so, whether they can influence long-term decision-making. We next assessed whether the associations learned in our task could be altered by an additional phase of subliminal learning where the reward/punishment contingencies were altered, such that the stimuli were no longer discriminatory. Finally, we probed the question of whether they undergo reconsolidation, by examining if application of this contingency shift during the hypothetical reconsolidation window following memory reactivation is more efficacious in altering instrumental task responses and preferences than the manipulation with no reactivation.

## Materials and methods

### Participants

Forty four participants (28 female, 16 male; mean age of 25.1 ± 3.4 years) were recruited from the Weizmann Institute of Science and the Faculty of Agriculture of the Hebrew University, Rehovot. Four participants were excluded from the analyses; two because they performed significantly above chance in the perceptual discrimination and or recognition tasks, and another two for constantly making a “Go,” “No-Go” or fixed alternate response in all trials of one of the testing sessions (see below). There remained 19 participants in the reconsolidation group and 21 in the control group. The experimental protocol was approved by the Institutional Review Board of the Sourasky Medical Center, Tel-Aviv and written informed consent was obtained from all participants.

### Experimental procedure

#### Overview

**Figure 1:**
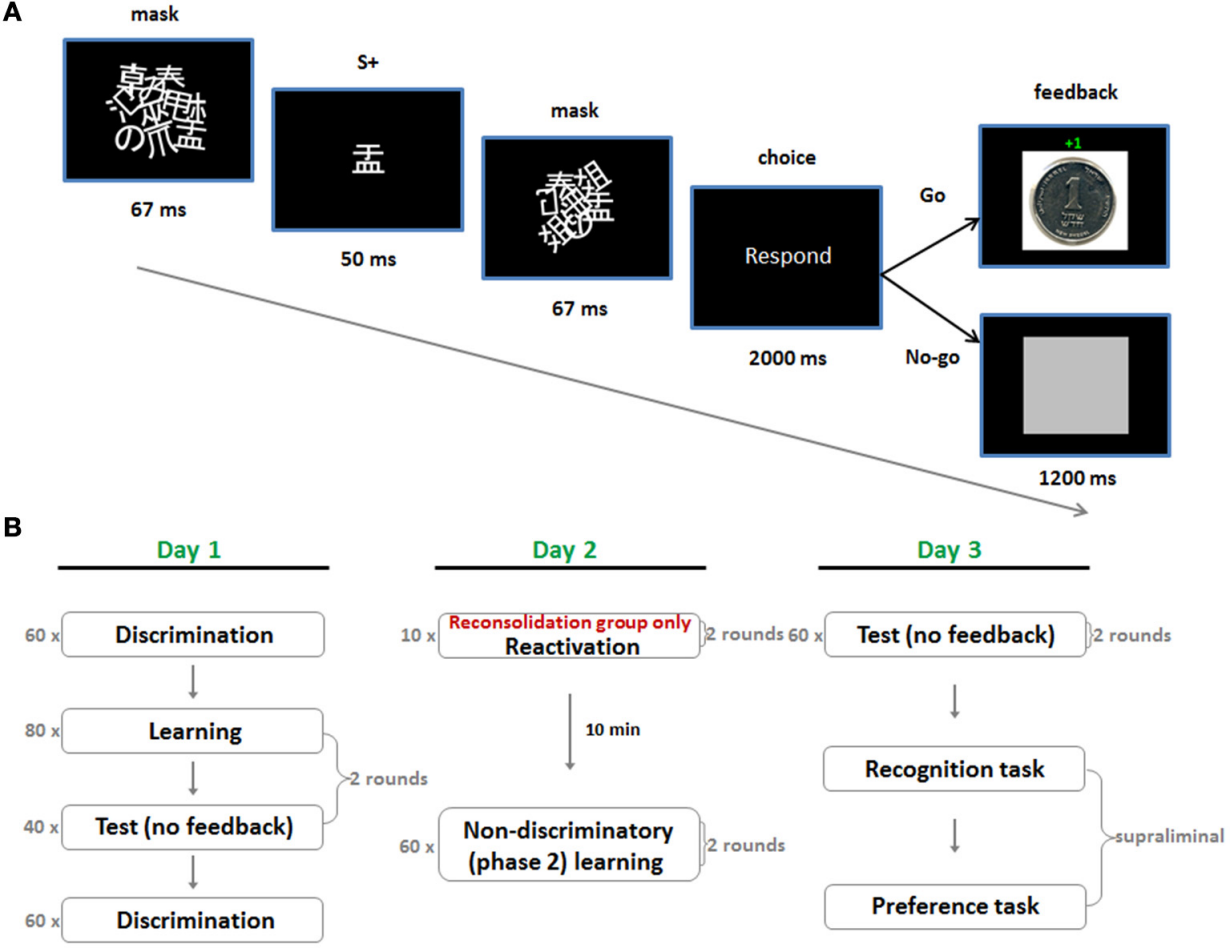
**Subliminal conditioning procedure and experimental protocol. (A)** A single trial of the subliminal, discriminated instrumental conditioning task used in acquisition on day 1. The S + depicted in this trial is appetitive (S + app) since a “Go” response always led to monetary gain following its presentation. **(B)** In test trials no feedback was provided but money could still be won or lost whereas in the non-discriminatory phase 2 trials a “Go” response led to a 50/50 win/loss outcome for all S + s. The critical manipulation in the reconsolidation group was reactivation prior to phase 2 learning on day 2.

The experiment took place over three consecutive days. On day 1 we employed a subliminal instrumental conditioning procedure which utilized discriminative stimuli (S^D^s) for reward and punishment (day 1; Figure 1A). This comprised rapid masked presentations of Japanese characters, which acted as appetitive (S + app) and aversive (S + av) discriminative stimuli, by way of a subsequent instrumental “Go/No-Go” response. A “Go” response led to a small monetary gain following the S + app and an equivalent loss following the S + av. A “No-Go” response led to a neutral outcome in both cases. Subjects were instructed to rely on their gut feeling to make as much money as possible by responding appropriately to the stimuli. Following learning there was a test session where the same stimuli were presented but no feedback was provided after the choice—though still playing for money. On day 2, subjects underwent a new phase of learning (phase 2) which entailed additional learning trials under non-differential reward/punishment contingencies (i.e., the stimuli were rendered non-discriminatory) (Figure 1B). In this phase the stimulus-response-outcome contingencies were altered by pairing each of the stimuli with a 50/50 win/lose outcome for the “Go” response. This procedure differs from extinction learning where the US is simply omitted, and reversal, where contingencies are entirely switched. In the reconsolidation group, phase 2 learning took place 10 min after reactivation by way of five test trials for each S+ (with monetary outcomes but no feedback), whereas in the control group it took place 10 min after entering the testing room but without reactivation. Affective evaluations of the stimuli were gaged with a supraliminal preference task, conducted following a further test session on day 3. In this task subjects were required to make binary choices between all possible combinations of S+ app, S + av and neutral stimuli (S−), according to their preferences. The subliminal nature of acquisition, reactivation and phase 2 manipulation was crucial since declarative knowledge strongly influences higher order behavior and it is unlikely that the phase 2 non-differential contingencies would have erased declarative knowledge of the contingencies on day 1 in either group, leaving preference to be determined by some reckoning of what was experienced over the different sessions (i.e., the various stimulus-reward/punishment contingencies).

#### Day 1

On day 1, participants were randomly assigned to one of two conditions—control or reconsolidation. Six stimuli taken from a set of Japanese characters (matched for size and complexity) were then randomly ordered to form three pairs of stimuli that were assigned to the subject for all 3 days: S + app, S + av (1st pair); S + app, S + av (2nd pair); S−1, S−2. The same six characters (randomized) were used for all subjects.

Discriminated instrumental conditioning (phase 1 learning) was implemented subliminally using a technique similar to Pessiglione et al. (2008) (Figure 1A). Each trial of learning started with masked presentation of a S+ on a PC. Mask 1 was first presented for 67 ms, followed by the S+ for 50 ms, followed by mask 2 for 67 ms. Masks 1 and 2 differed and comprised roughly 10 overlapping and rotated Japanese characters. These masks were identical for all subjects and remained the same throughout the experiment. Following presentation of the S+ subjects were cued to make a response. This phase lasted 2 s during which they could make a “Go” response (pressing the space bar) or a “No-Go” response (not pressing the space bar). In all learning trials, a “No-Go” response was followed by presentation of a neutral outcome—a gray square—whereas the outcome of the “Go” response depended on the preceding S+. If the appetitive S+ (S + app) was presented, a “Go” response was followed by a picture indicating they had won one shekel (≈25ȼ), whereas if the aversive S+ (S + av) was presented, it was followed by a picture indicating they had lost one shekel (see Supplementary information for task instructions provided to subjects). Note that any task with an instrumental contingency between stimulus, response, and outcome (S-R-O) also contains within it a Pavlovian type contingency between stimulus and outcome (S-O) and consequently, behavior can be influenced by a number of possible associations (Rescorla and Solomon, 1967; Mackintosh, 1983; Colwill and Rescorla, 1988).

Test trials were identical to learning trials except that no feedback was provided following responses—the subsequent trial began immediately following the 2 s response period. Subjects could still win or lose money during these trials and were aware of this.

In each round there were 80 trials of learning comprising 40 randomized presentations of each S+. Immediately following learning there were an additional 40 test trials (20 randomized presentations of each S+). There were two rounds of learning and testing, corresponding to the two pairs of S + s assigned to each subject. Learning of the second pair followed testing of the first and subjects were alerted between transitions from learning to testing and between rounds (Figure 1B).

**Figure 2:**
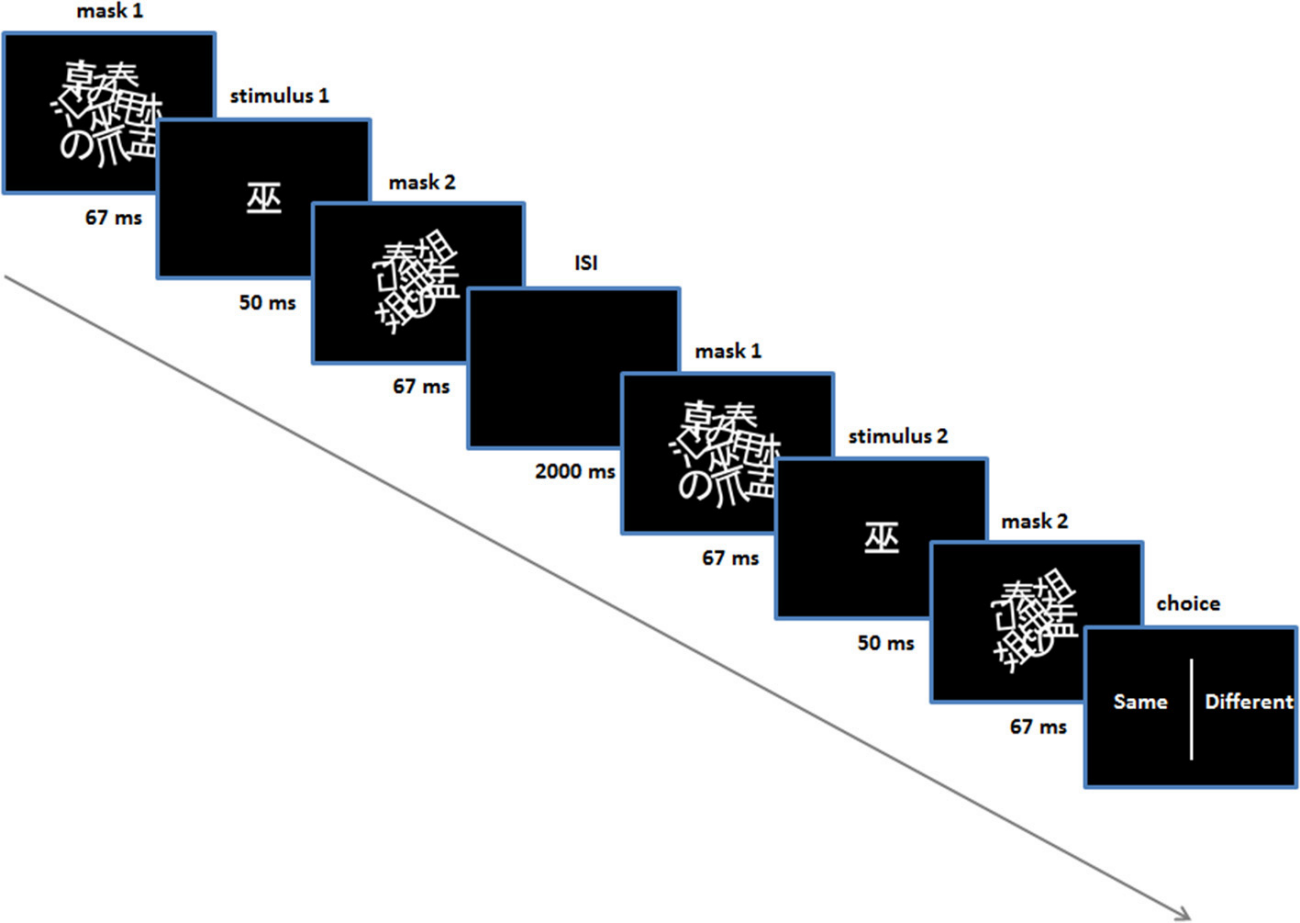
**Perceptual discrimination task**. Performed prior to and post-acquisition on day 1. Shown is one trial (in this case a “same” trial).

In addition, subjects were given perceptual discrimination tasks, prior to and following the conditioning procedure (Figure 1B)—the purpose of these tasks was to control for any conscious ability to discriminate stimuli. In these trials two stimuli were presented sequentially with an inter-stimulus interval of 2 s. They were presented in exactly the same manner as in the conditioning and test trials, using the same masks (Figure 2). For these trials an additional pair of Japanese characters were selected (i.e., not used in conditioning or subsequent tasks) and were identical for all subjects and in the pre and post-conditioning sessions. An algorithm selected one of the two stimuli randomly for each stimulus presentation in each trial (leading to four possible trial types: stimulus 1 (same), stimulus 2 (same), stimulus 1-then-2 (different), stimulus 2-then-1 (different). Following the second stimulus, a choice presented on the screen prompted the subject to indicate whether they thought the two stimuli were the same or different, using either the left or right shift key. Subjects' response prompted the next trial to begin. There was no feedback provided on their responses and no monetary incentive was offered for correct/incorrect answers (i.e., no reinforcement) (Figure 2).

Each of the discrimination tasks comprised 60 trials. In the pre-conditioning task, the stimulus duration was set at 50 ms (i.e., between the masks). Following these 60 trials a binomial test was automatically performed to assess whether accuracy for the subject was significantly above chance—if so, another 60 discrimination trials were performed where the stimulus duration was set to 33 ms. The purpose here was to set the stimulus duration for all subsequent procedures throughout the experiment, for each subject. In practice, no subject was able to discriminate above chance with a 50 ms stimulus duration, in the pre-conditioning test.

A short practice session of discrimination, learning and test trials was provided before the first discrimination task (utilizing additional characters that did not appear in any subsequent tasks). Subjects were debriefed at the end of testing regarding how well they thought they had done, if they thought they had learned anything and what they could describe about the stimuli.

#### Day 2

On day two subjects returned to the testing room for the phase 2 learning procedure. These trials were identical to the learning trials on day 1 except here a “Go” response led to a 50/50 win/loss outcome (i.e., irrespective of the S+)—that is the S + s were now rendered non-differential/discriminatory with respect to their reward and punishment contingencies. A round of phase 2 learning comprised 60 trials (30 randomized presentations of each S+) and each subject underwent two rounds. The S + app and S + av pairs used in each round were the same S+ pairs that were used for that subject in acquisition rounds 1 and 2 on day 1. The reminder session (for the reconsolidation group) comprised 20 test trials (i.e., no feedback, but playing for money) lasting less than 2 min. Each S+ from day 1 was tested five times successively.

The same instructions used for the learning task on day 1 were provided for the phase 2 trials. It was not specified if the stimuli were the same or different to those used in the prior day's trials. Subjects assigned to the reconsolidation condition (the reconsolidation group) underwent a reminder session when they entered the room, waited 10 min and then proceeded to the phase 2 trials. The purpose of these reminders was to reactivate the learned associations from day 1 and hence open the hypothetical reconsolidation window. Reconsolidation subjects were told that there was a short test of what they had learned on the previous day and that they could still win/lose on those test trials. Control group subjects did not undergo reactivation and started the phase 2 trials following a 10 min waiting period upon entering the testing room (Figure 1B).

#### Day 3

On day 3 subjects returned to the testing room where they were given three separate tasks. The first task comprised test trials that were identical to those on day 1 (as well as to the reminders on day 2 for the reconsolidation subjects). As on day 1, no feedback was provided but the subjects could still win or lose money with “Go” responses. There were two rounds (testing each subject specific pair of S + s conditioned on day 1 and rendered non-discriminatory on day 2), each comprising 80 trials (40 randomized presentations of each S+).

The remaining tasks on day 3 were supraliminal in nature. The second task was a recognition test where each of the six stimuli—S + app, S + av and S− (two of each)—was presented individually for 3.5 s. The S − s were neutral (novel) stimuli determined randomly for each subject on day 1 from the initial set of six characters but had not been presented in any of the preceding tasks. In order to present an equal number of “seen” and “unseen” stimuli we also included two additional neutral stimuli at this stage. Subjects were instructed to press the space bar if they thought they had seen the symbol in any of the sessions on days 1–3. The order of presentation was randomized for each subject. As with the perceptual discrimination task, the recognition test served to control for the formation of conscious S+ representations during subliminal sessions.

The final task was a supraliminal preference task. Here, subjects were given the instruction “choose the symbol you prefer” and subsequently made 15 binary choices. These choices were all possible combinations, randomized, of each of the 6 stimuli: 4 × S + app vs. S + av; 4 × S + app vs. S−; 4 × S + av vs. S−; 1 × S + app vs. S + app; 1 × S + av vs. S + av; 1 × S− vs. S−. Both options were simultaneously presented on the screen, separated by a perpendicular line and remained until the choice was made. Subjects chose between the stimuli on the left or the right of the screen using the left and right shift keys and the choices were self-paced.

At the conclusion of testing subjects were told how much money they had won or lost over the 3 days. This was summed to the 100 shekel payment for taking part and awarded to the subject.

All tasks were performed on a PC using the cogent toolbox for Matlab (www.vislab.ucl.ac.uk/cogent.php).

### Data analysis

All statistical tests were two-tailed and performed using Matlab and the Statistics toolbox. Chi-square tests were performed by hand. All means are reported ± s.e.m. in the Results Section.

#### Percentage correct instrumental responses

Percentage of correct instrumental responses were calculated by summing the number of “Go” responses following appetitive conditioned stimuli with the number of “No-Go” responses following aversive conditioned stimuli and dividing by the total number of trials for each subject individually. This was performed separately for learning and test trials on day 1, phase 2 learning trials on day 2 and test trials on day 3. To test whether performance differed from chance, these values were compared to a value equal to 50% of the number of trials, by means of one sample *t*-tests. Additionally, this measure was calculated for each of the two rounds individually (i.e., two pairs of S + s), and for the first and second half of trials for learning on day 1 (i.e., first 40 trials and second 40 trials) as well as individual round/half combinations (pair 1 half 1, pair 1 half 2, pair 2 half 1, pair 2 half 2) to assess how performance progressed with training. When comparing scores on different rounds/halves, we used within subjects paired *t*-tests. For between group comparisons, the above measures were compared using two sample *t*-tests.

#### Trial-by-trial percentage “Go” responses

Trial-by-trial responses were analyzed by summing the number of “Go” responses for each individual trial over all subjects and dividing by the number of subjects—that is the proportion of “Go” responses made by the group as a whole on each trial. Each S+ type (S + app and S + av) was analyzed separately in this manner for each of the instrumental tasks on days 1–3. This measure was calculated for each of the two rounds and then averaged. Additional analyses were performed for rounds 1 and 2 separately (day 1 tasks), and for each group separately. To assess the relationship between percentage “Go” responses and trial number, linear regressions were performed (with trial modeled as the predictor variable and percentage “Go” responses as the dependent variable) for each S + type, to obtain a measure of the slope (β) and the significance of the regression. Analyses of covariance (ANCOVAs) were performed to test for significant differences in the slopes and intercepts of the two regression lines (S + app vs. S + av), i.e., for main effects of trial and S+, as well as trial × S+ interactions. Since this method does not take into account inter-subject variability and trades this off for inter-trial variability we performed an additional ANCOVA, this time entering each subject individually into the analysis but binning their responses into eight 5-trial blocks (for each S+ individually) and determining a % Go response for each block. This method overcomes the problem of calculating trial-by-trial percentages for binary data by sacrificing a little trial-by-trial variance. To test for group differences in trial-by-trial performance we performed ANCOVAs on linear regression lines modeling the differential percentage “Go” response to the S + app vs. S + av over trials (i.e., S + app-S + av), for each group. In a stricter analysis we also compared each S+ specific regression line across the groups using ANCOVAs—i.e., the S + app vs. S + app regression lines and S + av vs. S + av regression lines in control and reconsolidation groups, overall, as well as for each round separately.

#### Perceptual discrimination

Responses in the discrimination task were classed as correct same, correct different, incorrect same and incorrect different. The number of correct “same” and correct “different” responses was summed for each subject. A binomial test was performed on this score to assess performance relative to chance. We analyzed the post-testing round in a similar manner. Any subject whose performance differed from chance was removed from the analyses. We also tested group performance for each group by comparing the subjects' scores with chance using a one sample *t*-test.

#### Recognition

For each subject the number of recognition responses to the S + s and the novel stimuli were summed separately (each giving rise to a number between 0 and 4). These scores were compared using a Wilcoxon matched-pairs signed-rank test, for each group separately. We compared the difference scores of recognized S + s minus recognized neutral stimuli across groups using the Wilcoxon rank-sum test.

#### Supraliminal preference

Choices from the preference task were grouped into three categories for each group: S + app vs. S−, S + av vs. S−, and S + app vs. S + av (four of each). Three choices were discarded from analyses (S + app vs. S + app, S + av vs. S + av, and S− vs. S−) since they provided no information on preferences between stimulus types. In an initial analysis we summed the number of choices of S + app, S + av and S− for each subject, over all the choices, to obtain an overall measure of preference. A repeated measures ANOVA was used to test for any differences in overall preferences to the stimuli. This was performed for each group separately, followed by *post-hoc* comparisons (Tukey's HSD) in the case of a significant result. We also directly compared overall preference scores to each stimulus across groups using two-sample *t*-tests. The null hypothesis in these initial comparisons was that all stimuli should be equally preferred, however, since these overall preference measures blended scores from three different choice categories, we also analyzed the preferences in each choice category individually. As these data were not normally distributed non-parametric tests were used. We first focused on the S + app vs. S + av choices and performed a chi-square test on the group summed scores for each option chosen, to assess whether preference differed from chance in each group. Another 2 × 2 chi-square directly compared these scores across groups. To assess whether preference in the control group was driven by attraction to the S + app or aversion to the S + av, we performed chi-square tests on the remaining choice categories.

## Results

### Day 1

#### Instrumental learning—percentage correct

**Figure 3:**
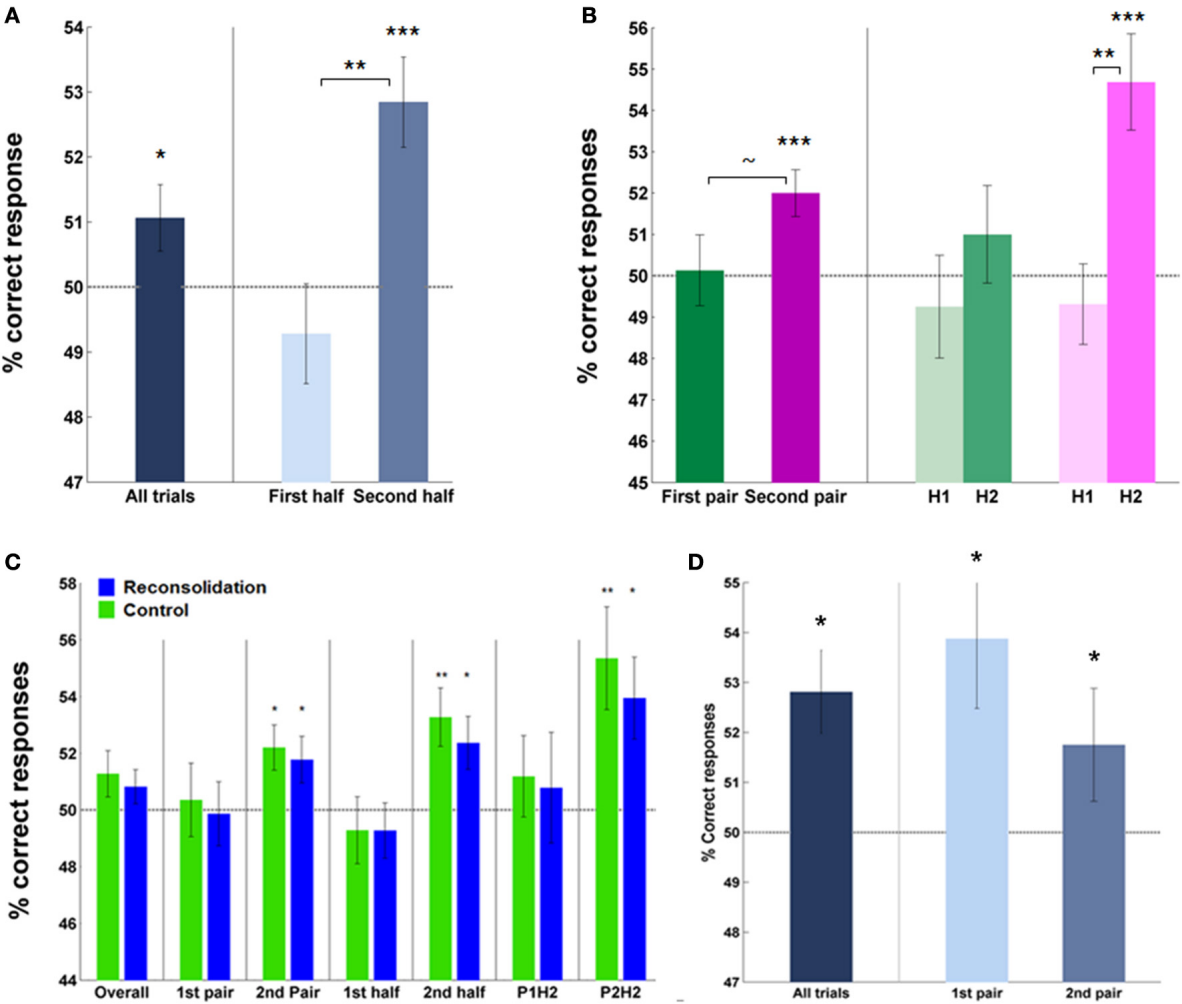
**Instrumental performance during acquisition (day 1)—percentage of correct responses. (A)** Subjects (overall) performed significantly above chance, particularly in later trials, indicating successful learning of cue-response-outcome contingencies. **(B)** Subjects' performance also improved in the second round (pair) of learning compared to the first and within each pair, over trials (on the right). Note, trial-by-trial analyses revealed a significant effect of learning in the first round (Supplementary Figure 1). **(C)** The groups did not differ in percentage correct responses during acquisition—overall or on specific rounds or halves. Each group performed above chance when analyzing the percentage correct scores during the second half of trials and also on the second round. **(D)** Performance in test trials was significantly above chance in both rounds. (Asterisks above bars indicate significance vs. chance, those between bars are within subject comparisons across halves or rounds; ~, trend, ^*^P < 0.05, ^**^P < 0.01, ^***^P < 0.001. Bars and error bars represent mean ± s.e.m.).

Analysis of the percentage correct instrumental responses (“Go” responses to S + app, “No-Go” responses to S + av) vs. chance over all subjects, trials and rounds, revealed a significant effect of acquisition on day 1 (mean = 51.40 ± 0.51%, *p* < 0.05; Figure 3A). This score was higher in the second half—the latter 20 trials—(mean = 52.84 ± 0.69%, correct vs. chance; *p* < 0.0005) compared to first half of trials (mean = 49.28 ± 0.77%, second vs. first half; *p* < 0.005), and also in the second pair of S + s (second round) (mean = 52.0 ± 0.56%, correct vs. chance; *p* = 0.001) relative to the first (mean = 50.13 ± 0.86%, second vs. first round; *p* = 0.077), indicating an effect of learning over trials and rounds (Figure 3B). No significant difference between groups was observed in this measure, overall, by half or by pair (Figure 3C).

Note that this measure of performance combined learning from the S + app and S + av, essentially measuring the differential “Go” and “No-Go” responses to the stimuli. Analysis of percentage correct responses to each S+ *individually* is uninformative here since subjects made significantly more “Go” responses overall (i.e., leading to the appearance of many correct responses to the S + app and incorrect responses to the S + av).

#### Instrumental learning—trial-by-trial responses

**Figure 4:**
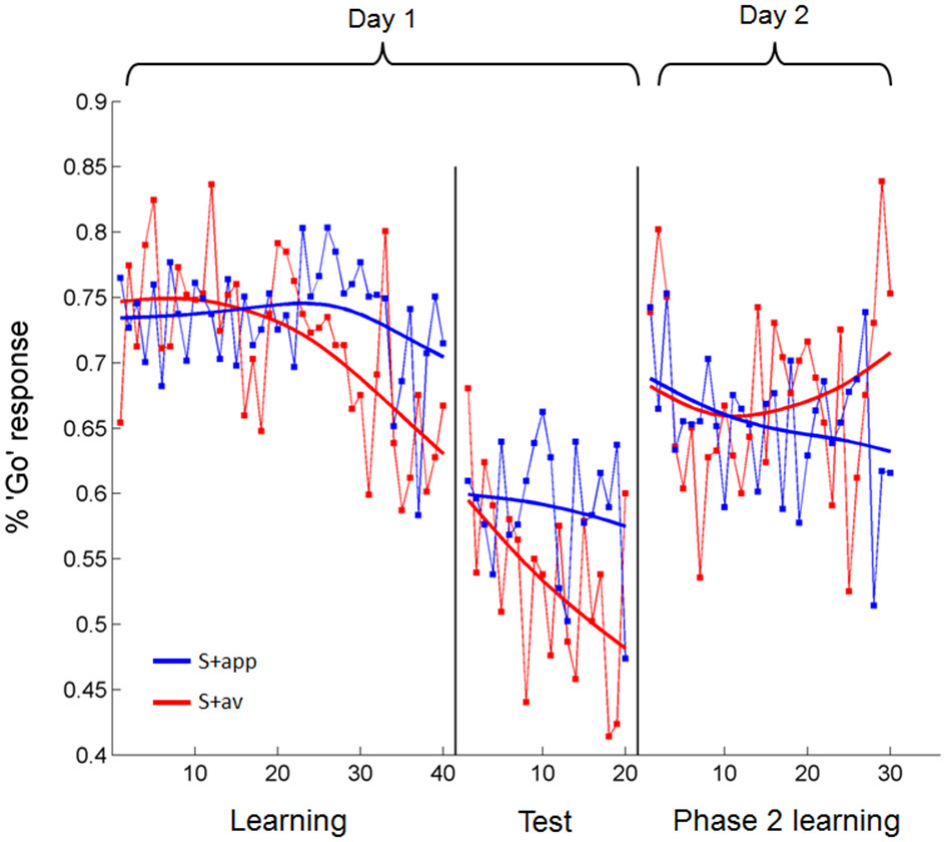
**Trial-by-trial percentage “Go” responses to the appetitive and aversive stimuli over days 1 and 2**. During acquisition subjects learned to make fewer “Go” responses to the S + av than the S + app as trials progressed. In test trials, where no feedback was provided but subjects were still playing for money, “Go” responses to the S + app remained higher than to the S + av. This difference was abolished on Day 2. All statistical analyses were based on linear regressions. Data presented are from all subjects averaged over rounds. Fitted models (smoothing spline) are presented for display purposes.

In a more sensitive analysis of the acquisition blocks we looked at the trial-by-trial change in the percentage of subjects' “Go” responses to the S + app and S + av individually, modeling these with linear regressions (Figure 4). An analysis of covariance (ANCOVA) of these regression lines revealed a main effect of S+ [S + app > S + av; *F*_(1, 76)_ = 3.99, *p* < 0.05], matching the percentage correct analyses, and a significant S+ × trial interaction [*F*_(1, 76)_ = 7.93, *p* < 0.01]. The latter result shows that the divergence of the regression lines—i.e., difference in percentage “Go” responses to S + app vs. S + av—significantly increased over trials, consistent with a learning effect. We also demonstrated this interaction [*F*_(1, 636)_ = 4.1, *p* < 0.05] in an additional ANCOVA where each subject's responses to each S+ were binned into blocks of 5 trials to calculate a percentage “Go” response for each block, and entered separately into the analysis (see methods). Interestingly, although the slope of S + av regression line was significantly negative (β = −0.31, *p* = 0.0001), reflecting a diminishing number of “Go” responses over trials, the slope of the S + app regression was not significantly different from zero (Figure 4). This would suggest that since the proportion of “Go” responses were very high to begin with—presumably resulting from subjects' initial desire for feedback in order to become able to distinguish the stimuli—instrumental learning of the S + app was concealed by the initial (artificially) high level of correct responses, which was likely unsurpassable given the subliminal presentation. As subjects learned that indiscriminately high rates of “Go” responding was suboptimal, learning to the S + app may have been manifested instead as a resistance to this downward shift in “Go” responses over trials (Figure 4). The increased sensitivity of the trial-by-trial analysis revealed that instrumental learning also took place in the first round (pair of S + s), which was not apparent in the percentage correct analysis (Supplementary Figure 1). To compare groups we performed an ANCOVA of regressions modeling the differential percentage of “Go” responses to the S + s over trials in each group (Supplementary Figure 1). This revealed no group difference in trial-by-trial degree or rate of learning; nor did additional ANCOVAs directly comparing the S+ specific regressions across groups (see Supplementary Figure 1 for further group and round-specific analyses). Our demonstration of subliminal instrumental conditioning thus replicates an earlier and similar study (Pessiglione et al., 2008) albeit with differences in the degree and symmetry of the effect in relation to aversive and appetitive cues.

#### Test trials

Successful instrumental conditioning was also evident from subsequent test sessions where subjects were still playing for money but no feedback was provided, both from the percentage correct responses vs. chance analysis (mean = 52.81 ± 1.07%, *p* < 0.025) and from a main effect of S+ in the trial-by-trial ANCOVA (S + app > S + av; *F*_(1, 36)_ = 9.85, *p* < 0.005; Figure 4). Here, the Trial × S+ interaction did not reach statistical significance, consistent with the fact that no feedback was provided in these trials and hence no additional learning was expected to take place (however, performance did appear to improve to some extent—this may be explained by poor performance at the start of test trial rounds, where subjects reported the lack of feedback to be disconcerting). Again, no group differences were apparent in test trial performance either in percentage correct scores (control mean = 53.1 ± 1.66%; reconsolidation mean = 52.5 ± 1.35%) or in trial-by-trial responses (Supplementary Figure 2). Over all subjects, test trial performance was slightly better in round 1 than round 2—both rounds being significantly greater than chance (Figure 3D) and showing a main effect of S+ in the trial-by-trial ANCOVA. Group wise, test trial performance was better in round 2 for the control subjects and round 1 for the reconsolidation subjects; these group differences were not significant.

#### Self report and discrimination trials

Importantly, conditioning took place without explicit awareness. This was evident from introspective reports during debriefs on day 1. Most subjects were not certain/did not believe there were any differences in the stimuli they saw and could not accurately describe what they looked like—in keeping with previous accounts of this masking technique (Marcel, 1983; Esteves and Öhman, 1993; Kim and Blake, 2005; Pessiglione et al., 2008). In almost all cases subjects were unaware of how well they had performed and often reported that their responses were based on guesses. Some subjects “felt” there were differences in the stimuli but in no cases did they correctly describe their discriminatory features. This was also confirmed by chance level performance of perceptual discrimination tasks prior to learning and post-testing on day 1. In the pre-acquisition round, no subject differed from chance-level performance with a stimulus duration of 50 ms. The individual binomial tests showed that two subjects performed significantly above chance in the post-testing round—these subjects were removed from all analyses. Group performance in the post-testing round revealed no significant difference from chance-level discrimination performance in either group (control mean = 51.07 ± 1.23%; reconsolidation mean = 49.74 ± 1.25%).

#### Day 3 recognition

Furthermore, in the supraliminal recognition task on day 3, comparison of correctly recognized S + s with incorrectly recognized (i.e., recognized novel) stimuli (S − s) revealed no significant difference in the control group (mean S+ = 57.14 ± 6.49%; mean novel = 55.95 ± 5.95%) and in the reconsolidation group (mean S+ = 57.89 ± 6.36%; mean novel = 48.68 ± 5.57%). There was no significant difference in the group recognized S+ minus recognized novel scores. Thus, these three evaluations suggest it is unlikely that subjects formed conscious representations of cue-outcome associations. Subject debriefs indicated that performance improved in the second round because subjects learned to better rely on their “gut feeling” or intuition, and realized that other strategies (for example focusing intently on one point of the screen, or trying to infer a (nonexistent) pattern of reinforcement) did not help. The ability to forego the tendency to try and explicitly unveil the stimuli and their associations with the outcomes—and instead make what seems like arbitrary button presses—was initially unnatural for participants. Had the subjects habituated to the masking and become aware of the stimuli, performance would have been dramatically greater than chance.

### Day 2

#### Non-differential learning trials—percentage correct and trial-by-trial responses

Analysis of instrumental performance during phase 2 learning (day 2) showed that conditioned instrumental responses were abolished, both in terms of percentage correct responses (in relation to the original contingencies; mean = 48.08 ± 1.51%) and trial-by-trial changes in percentage “Go” responses for each S+ (Figure 4; no significant main effect of S+ or difference in slopes in the ANCOVA). When scored according to the contingencies presented on day 1, neither group differed significantly from chance-level, or from each other in their percentage correct responses (mean control = 48.17 ± 2.26%; mean reconsolidation = 47.98 ± 2.03%). Similarly, there were no group differences when comparing trial-by-trial responses (Supplementary Figure 3). The extent to which instrumental performance was degraded by these non-discriminatory trials, as opposed to intervening time, is questionable, since percentage “Go” responses to the S + app and S + av did not differ in early trials (Figure 4)—suggesting that the stimulus-action-outcome learning had degraded between days.

### Day 3

#### Preferences

**Figure 5:**
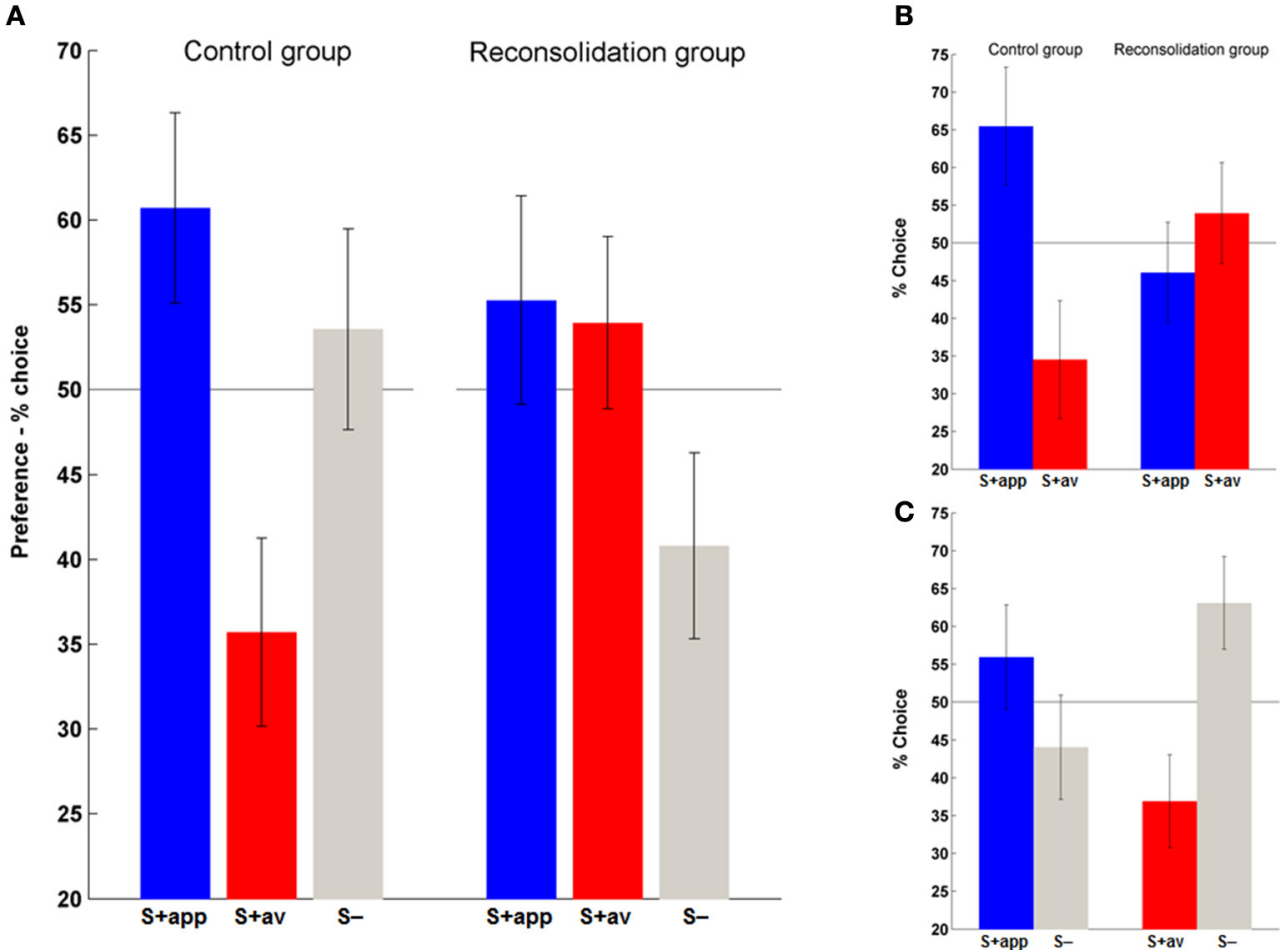
**Affective evaluation of stimuli. (A)** Overall preferences to the S + app, S + av and (neutral) S−. The distribution of preferences did not differ from chance in the reconsolidation group, whereas the S + app was chosen significantly more often than the S + av in the control group. **(B)** A similar pattern was evident when only considering S + app vs. S + av choices. **(C)** Choices between S+ and S− in the control group indicate that an aversion to the S + av was likely to be more significant than an attraction to the S + app in driving the S + app > S + av preference. (Bars and error bars represent mean ± s.e.m.).

Having established acquisition of instrumental conditioning on day 1, we next examined the persistence of the (affective) stimulus-outcome associations that would have presumably been formed during acquisition (Rescorla and Solomon, 1967; Mackintosh, 1983), and the efficacy of the contingency change in phase 2 learning in altering these associations, by assessing the conscious hedonic evaluation of stimuli. In this supraliminal preference task (day 3) we also introduced the neutral stimuli (S−) and asked subjects to choose their preferred symbol in binary choices of all stimulus combinations. We first assessed overall preferences for each stimulus type (S + app, S + av and S−) by summing the number of times they were chosen over all choices. Repeated measures ANOVAs revealed a significant difference in preferences in the control [*F*_(2, 40)_ = 3.41, *p* < 0.05] but not the reconsolidation group (Figure 5A). *Post-hoc* comparisons confirmed that the marked difference in preference to the S + app compared to the S + av was significant in the control group (*p* < 0.05; Figure 5A). Direct group comparisons of these overall scores also showed that the S + av was significantly less preferred in the control group (*p* < 0.05) with no significant group difference for the other stimuli.

In order to examine in more detail what was driving the preferences we also analyzed each choice type individually, first focusing on S + app vs. S + av decisions (Figure 5B). Here again, control group subjects significantly preferred the S + app, choosing them roughly twice as often as the S + av [χ^2^_(1)_ = 8.05, *p* < 0.01]. In contrast, the reconsolidation group was indifferent to the two options in this choice type. The S + app − S + av difference was also significant when directly comparing groups (i.e., interaction) [χ^2^_(1)_ = 6.12, *p* < 0.025].

Finally, to determine whether the preferences in the control group were driven by an attraction to the S + app or an aversion to the S + av (or both) we focused on each of the S + vs. S− decisions (Figure 5C). Here, in the S + av vs. S− choices there was a significant preference for the S− [χ^2^_(1)_ = 5.76, *p* < 0.025]. There was also a greater preference for the S + app vs. the S− although this difference was not significant. Interestingly, the latter finding dovetails with the instrumental conditioning which also appeared to be more driven by the S + av (however a meaningful direct comparison (e.g., by comparing slopes of trial-by-trial “Go” responses) was precluded because of the initial very high rates of “Go” responses and possible ceiling effect with regards the S + app). This superior learning from the aversive outcome may relate to the fact that a given loss is perceived to be more aversive than an equivalent gain is rewarding (loss aversion—Tversky and Kahneman, 1992). On the other hand, the degree of instrumental learning is not prima facie correlated with the degree of stimulus-outcome learning/preference for each stimulus in this task. Thus, the difference in preferences in the control group was slightly greater in the first pair of stimuli (1st round) even though the instrumental performance was better in the second round (Figure 3 and Supplementary Figure 1)—preferences across all choices in the control group were 62% (S + app) and 31% (S + av) for the first pair of S + s, and for the second pair 60% (S + app) and 40% (S + av). Corresponding data for the reconsolidation group are 61% (S + app) and 54% (S + av) (1st pair) and 50% (S + app) and 54% (S + av) (2nd pair). Note that to control for their aesthetic properties, all stimuli were randomized across subjects.

#### Test trials (instrumental)

No evidence of conditioned instrumental responses was apparent in either group on day 3 test trials preceding the preference task. When scored according to the contingencies on day 1, the percentage correct responses in test trials on day 3 did not differ significantly from chance in either group (mean control = 51.28 ± 1.59%; mean reconsolidation = 49.54 ± 0.83%) or differ between groups. In a trial-by-trial analysis of these trials ANCOVAS revealed no significant difference in “GO” responses to the S + s in either group—nor were there any between group differences. Lack of persistence (or recovery) of instrumental responses indicates that the conditioned preferences revealed in the preference task were driven by affective properties of the S + s (i.e., stimulus-outcome associations) and did not result from any instrumentally conditioned responding. In addition the nature of responses in the two tasks (“Go/No-Go” vs. Left/Right) differed, thereby precluding this interpretation.

## Discussion

Recent advances in the understanding of emotionally driven learning have focused on the physiological or neurological responses evoked by conditioned stimuli. Conversely, the characterization of the higher order affective and motivational properties acquired by stimuli during conditioning has received less attention. Yet determining the characteristics of such associations is paramount, because they allow environmental stimuli to profoundly influence volitional behavior and decision-making by initiating desires and aversions, guiding action selection and controlling behavioral vigor (Rozin et al., 1998; De Houwer et al., 2001; Cardinal et al., 2002; Dickinson and Balleine, 2002; Berridge, 2004). The acquisition of likes and dislikes is sometimes referred to as evaluative conditioning (EC) (Rozin et al., 1998; De Houwer et al., 2001; Hofmann et al., 2010), although the paradigm used here differs from typical EC procedures. EC is hypothesized to be a feature of classical conditioning whereby the CS forms an association with the valence of the US and is thought to be one of the fundamental mechanisms by which preferences are acquired through experience—often effectively exploited as a technique in advertising by pairing products with positively valenced stimuli (Gorn, 1982; Kim et al., 1996; Gibson, 2008; Sweldens et al., 2010). Such representations play a central role in the guidance of actions based on future reward/punishment—a basic form of decision-making (O'Doherty et al., 2006). Interestingly, EC has on occasion been shown to resist extinction learning (Baeyens et al., 1988, 2005a,b; De Houwer et al., 2001; Vansteenwegen et al., 2006; Dwyer et al., 2009; Hofmann et al., 2010), though these were supraliminal studies and the preferences may have reflected declarative memory of the original contingencies. We show that human preferences can be acquired subliminally and still influence long-term choice behavior. By avoiding the formation of declarative knowledge during acquisition we provide a critical step in understanding the neuropsychological basis of likes and dislikes. Our results highlight the importance of initial experiences in the learning of preferences and suggest that the characteristics of their underlying associations can markedly differ from some typically studied emotionally learned responses. They are both long lasting and resilient. These features may explain the remarkable persistence of many learned human preferences, such as food tastes (Dwyer et al., 2009), phobias (Rozin et al., 1998), and brand attitudes as well as the efficacy of advertising in shaping consumer choice (Sweldens et al., 2010)—even when individuals are not aware of the learning experience or have forgotten it.

The necessity of conscious awareness in human classical conditioning has been strongly debated (Lovibond and Shanks, 2002; Wiens and Öhman, 2002). In a recent study which was designed to address this question, autonomic (skin conductance) responses to a stimulus associated with an electric shock were acquired non-consciously but attenuated extremely rapidly (Raio et al., 2012)—within the acquisition session itself. An additional experiment in this study also revealed no evidence of conditioned responses (CRs) during a test session on a subsequent day—following a shorter acquisition period where conditioning was terminated prior to attenuation. In light of this, the conditioned preferences to the stimuli exhibited by the control group here are striking, both in their magnitude and their persistence over days following acquisition—enduring the phase 2 manipulation (which was designed to abolish them) and multiple test rounds. Subliminally acquired preferences have been previously demonstrated immediately following learning; once in a similar masked instrumental conditioning procedure (Pessiglione et al., 2008) and in evaluative conditioning paradigms (De Houwer et al., 2001; Hofmann et al., 2010)—though these have had limited success and have been criticized on a number of grounds (Field, 2000; Lovibond and Shanks, 2002). To our knowledge, this is the first demonstration of the persistence of such preferences beyond the immediate aftermath of acquisition, and their resistance to alteration.

We caution that there is substantial debate relating to the complexity of subliminal processing of information, and the methods used to achieve it (Maxwell and Davidson, 2004; Pessoa, 2005; Wiens, 2006). A critical issue upon which there is no consensus is the criteria used to determine how effectively a stimulus has been occluded from awareness, or indeed what exactly constitutes awareness. A distinction respected in the literature is between subjective criteria, sometimes termed *explicit awareness*, and objective criteria. The former assesses the phenomenological experience of the subject by way of self-report concerning the stimuli and the subject's task performance, whereas the latter typically assesses performance on forced-choice discrimination, or signal detection tasks. The absence of explicit awareness does not imply that the information is inaccessible through other measures (e.g., forced choice discrimination) which may or may not index other aspects of awareness, merely that an individual is unable to report conscious experience of the stimulus' existence or appearance—indeed, our study is premised on such an assumption. A number of concerns have been raised with regard to visual masking paradigms in particular because there is large individual variability in explicit awareness thresholds. Furthermore, even in explicitly unaware individuals, large differences have been demonstrated in perceptual thresholds to more sensitive measures (such as forced choice tasks) which may go unnoticed unless sufficiently sensitive tasks are used to assess the level of perceptual salience and processing of the stimuli on an individual basis (Maxwell and Davidson, 2004; Pessoa, 2005; Wiens, 2006). It is for this reason that we relied on three measures (comprising both objective and subjective/explicit assessments) to try to eliminate the possibility that subjects may have become consciously aware of the stimuli: forced-choice discrimination (pre and post-learning), recognition task on day 3, and self-report—all on a per-subject basis. Moreover, we tailored the duration of stimulus presentation to each individual's own performance on the pre-learning discrimination task. This number of awareness measures is very stringent in relation to other masking studies. Backward masked facial expressions are a priori likely to be more difficult to effectively occlude from awareness, since individuals have a lifetime's experience of processing facial expressions and therefore they are much more amenable to detection than the novel complex stimuli used in our task (Maxwell and Davidson, 2004). This may explain why few of the subjects were excluded based on above threshold performance, and that even at 50 ms presentations the majority were unaware of the stimuli, in contrast to some findings with emotional faces. However, it is possible that individuals would have differed with regards to their perceptual sensitivity outside explicit awareness had additional measures been acquired. For example, a discrimination task requiring subjects to identify a masked stimulus by way of a forced choice discrimination between two supraliminal stimuli may have been more sensitive to individual and group differences.

The duration of subliminal instrumental (stimulus-action-outcome) learning has not previously been addressed—to our knowledge—but appears to fall somewhere in the middle, persisting beyond acquisition (apparent in test trials) but not to the same extent as the (stimulus-outcome based) preferences. Thus, even at the start of the non-differential conditioning trials on Day 2, behavior did not appear to be under the control of the instrumental associations formed on Day 1. This was also the case with non-consciously acquired skin conductance responses in Raio et al. (2012), but in that study, the responses began to decline within the acquisition block and were not apparent at all by the end of the learning trials. Nor was there any strong indication of persistence of this learning on Day 3 test trials, in either group. This dissociation suggests that long-term consolidation of instrumental conditioning may be more dependent on conscious awareness than the affective responses supporting liking are, or that the extent to which perceptually degraded stimuli have access to these different aspects of cognition and their associated neural structures is not uniform, with a higher threshold required for the former. Alternatively, the higher order affective and motivational associations formed with the stimuli may be much more resilient and less prone to degradation over time, or with behavioral manipulation (such as extinction training), than either instrumental or more reflexive Pavlovian responding. This dissociation is commonly observed in animal models of addiction where effects such as conditioned reinforcement—the ability of Pavlovian cues to support new learning—persist for months without any further experience of drug administration, and are also resistant to extinction of the original stimulus-action-outcome learning, and devaluation of the US (Everitt and Robbins, 2005; Milton and Everitt, 2010); a strong factor leading to cue induced relapse during periods of withdrawal. The general Pavlovian description of preference formation posited in the EC literature does not necessarily imply that preferences resulting from our task are based on a similar, direct association between the stimulus and the affective properties of the US (outcome)—a number of other, less direct associations may be at play here. Given the dissociation between instrumental responses and preferences on day 3 however, it is a fair assumption that these responses were governed by separate associations.

Our results indicate that despite the strength of appetitive and aversive affective associations, they can also undergo reconsolidation. Existing human reconsolidation studies focus on classical fear conditioning, employing primary reinforcement (e.g., Schiller and Phelps, 2011). In that paradigm, a technique of post-reactivation extinction learning has been successful in preventing spontaneous recovery of autonomic skin responses (Schiller et al., 2010; Oyarzún et al., 2012) (but see Kindt and Soeter, 2013; Delamater and Westbrook, 2014, for counter examples). More recently this technique has been used in the appetitive domain to ameliorate the motivational salience of drug associated stimuli (Xue et al., 2012). Our result provides further evidence for the efficacy of post-reactivation manipulation of reconsolidation, perhaps even beyond the prevention of spontaneous recovery, but in altering associations that are strong enough to be resistant to extinction protocols in the first place. However, our paradigm differs in the nature of both reactivation and manipulation employed. The use of extinction trials as a means of reactivating memory prior to identical extinction trials invites the question as to whether the effect is engendered by spaced extinction rather than reconsolidation manipulation (Alberini, 2013). Here, the test trials used for reactivation differed from the phase 2 learning and did not lead to any degradation of the learned associations (as gaged by instrumental responses on day 1) prior to the phase 2 trials. Furthermore, the subliminal nature of the task also ensured that declarative knowledge played no part in the acquisition and alteration of the learned associations, thus eliminating a potential confounding variable (Schiller and Phelps, 2011).

These findings show that reconsolidation is a wider phenomenon than previously described, common to a number of forms of associative learning as well as learning driven by secondary reinforcement such as money, and can occur without awareness. Our results seem to counter a recent theory that new learning (or the generation of a prediction error) is required during reactivation in order to trigger reconsolidation (Pedreira et al., 2004; Morris et al., 2006; Díaz-Mataix et al., 2013; Sevenster et al., 2013). Here, no new learning took place during the reminder test trials since there was no feedback, consistent with other examples from the animal literature (Duvarci and Nader, 2004) where reconsolidation was triggered by identical trials involving no new learning, and a human study (Hupbach et al., 2008) showing that a novel environment does not trigger reconsolidation. The exact rules governing the updating processes during reconsolidation are not yet fully understood and there exist many counter examples to most boundary conditions (Alberini, 2013). Another possibility is that boundary conditions governing updating of certain forms of learning are different to others, such as human preferences. It is important to note that although an effect on reconsolidation may be one explanation of the group differences observed here, the critical difference was the reminder, and since there was no additional group with a reminder before the manipulation, or long after it, we were unable to conclusively conclude that a reconsolidation window was opened or what its duration was. Moreover, the validity of the reconsolidation window concept has recently come into question (Delamater and Westbrook, 2014) based on findings that reactivation treatment may work whether it occurs within the window or not, e.g., by ordering extinction training prior to the reminder (Baker et al., 2013; Millan et al., 2013; Stafford et al., 2013). Finally, we note that an alternative account of our results could be based on a difference in renewal of the learned associations on day 1 in the two groups. One could assume that the day 2 reminder trials (due to their similarity to (no-feedback) test trials on day 1) rendered the “context” of day 2 similar to that of day 1 for the reconsolidation group, but not for the control group. It could then be argued that the (no-feedback) test trials on day 3 activated the context of day 1 for the control group but not for the reconsolidation group—akin to an AAA vs. ABA renewal comparison for the reconsolidation and control groups respectively. Alternatively, the reminder instructions explicitly referencing the previous day's learning in the reconsolidation group, could have rendered this akin to an AAB vs. ABC context procedure, with the latter occasionally showing more renewal (e.g., Üngür and Lachnit, 2008).

Although higher order incentive learning aids in the procurement of rewards and avoidance of punishment, it can sometimes go awry—aberrant affective salience of environmental cues is critical in the maintenance of disorders such as addiction, post-traumatic stress disorder and phobias. For instance, in addiction, cues and contexts associated with drug taking are attractive and can induce immense cravings, hijacking behavior to seek drugs and leading to relapse (Berridge, 2004; Berridge and Aldridge, 2009; Milton and Everitt, 2010). We conclude that our data may support the use of reconsolidation-targeted treatments to overcome the long-lasting, maladaptive influences of these cues on human behavior.

### Conflict of interest statement

The authors declare that the research was conducted in the absence of any commercial or financial relationships that could be construed as a potential conflict of interest.
