# Supplementary material for: Unconscious learning of likes and dislikes is persistent, resilient, and reconsolidates
Source: Front Psychol. 2014 Oct 6;5:1051. doi: 10.3389/fpsyg.2014.01051 (PMC4186287; doi:10.3389/fpsyg.2014.01051)
Supplement: Supplementary file 1 [file Presentation1.PDF]

Supplementary information

Task instructions – Day 1

Subjects were provided with the following instructions:

The aim of this game is to win money by learning to make appropriate responses to cues.

At the beginning of each trial you must orient your gaze to the center of the screen and pay attention to a masked (hidden) cue which will appear very briefly. You will not be able to fully perceive the cue that is hidden behind the mask.

You will then see the word "respond", at which point you will have 2 seconds to make your choice between:

- Pressing the space bar
- Leaving the space bar

You will then see the outcome of your choice. Not pressing the space bar is safe – you will always get a neutral outcome (0 shekel). Pressing the space bar is risky – you can equally win 1 shekel or lose 1 shekel; this depends on whether a winning or losing cue was hidden behind the mask.

There is no logical rule to find in this game. You will see an equal number of winning and losing cues but the order will be random, therefore if you never press the key, or press it every trial your overall payoff will be zero. To win money you must press if you feel the cue is a winning cue and not press if you feel it's a losing cue. Your choices should improve with every trial by learning from the feedback and your unconscious emotional reactions. Just follow your gut feeling and you will win, and avoid losing, many shekels. You will be paid 100 shekel for taking part in this experiment and any money you win or lose in the task will be added to or taken away from this amount.

There will be two different rounds of this game - you will be notified when the new round begins. In addition, each round is divided into two phases: a 'learning' phase and a 'testing' phase. The learning phase is exactly as described above. In the testing phase however, you won't receive any feedback on your responses, but you will still win/lose money on these trials. You will be notified before these 'no-feedback' trials begin. At the end of the experiment, on the final day, we will tell you how much money you won/lost from the task.

Supplementary figures

All means are reported ± SEM. In trial-by-trial percentage 'Go' responses figures, fitted models (smoothing spline) are superimposed for display purposes.

Day 1

Acquisition

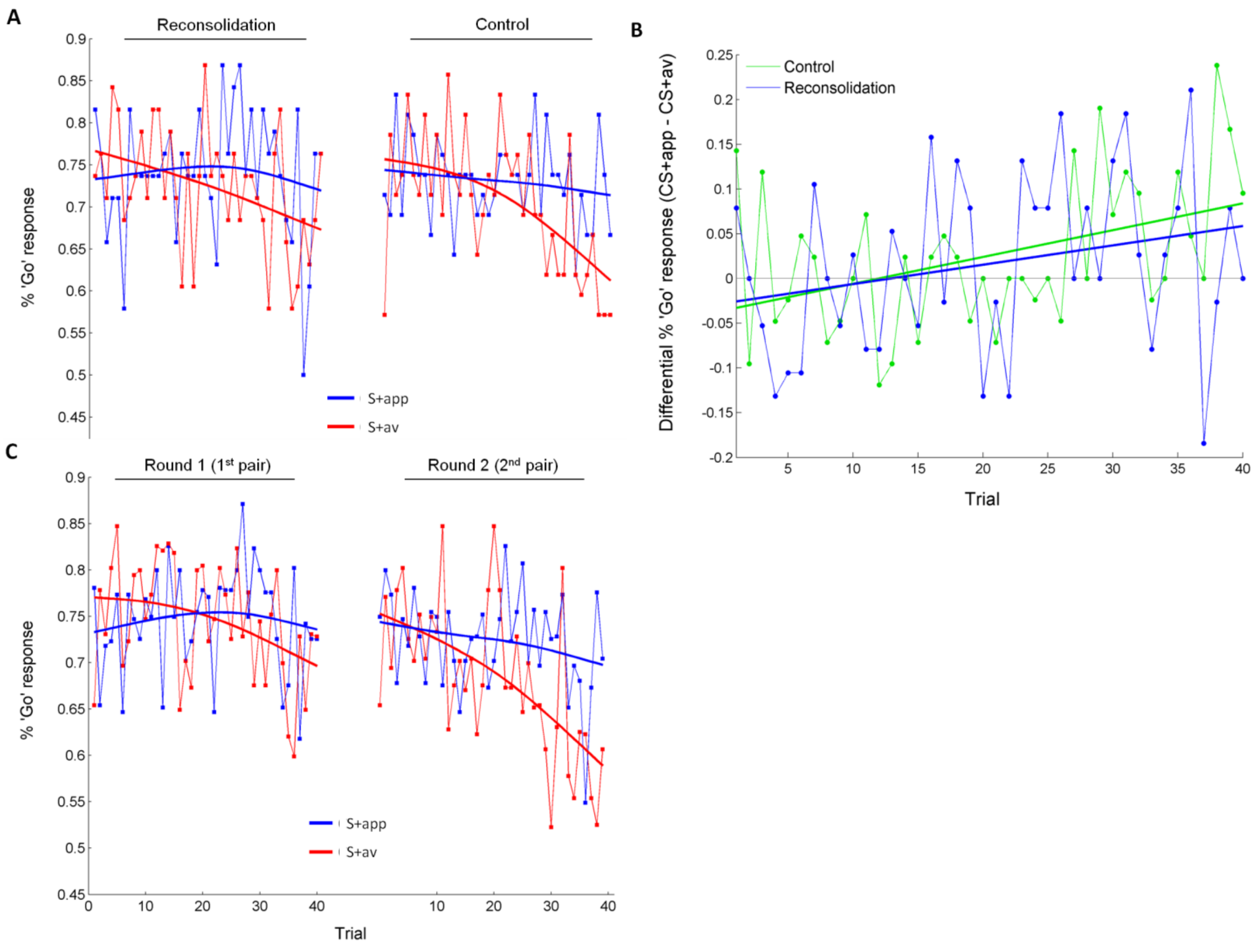

Supplementary Figure 1. Trial-by-trial percentage 'Go' responses to the S+app and S+av during acquisition.

(**A**) Percentage 'Go' responses to each S+ for each group separately (averaged over rounds). Linear regressions showed that in both reconsolidation (β = -0.2, P = 0.01) and control (β = -0.35, P < 0.001) groups, responses to the S+av decreased over trials, but the slopes of the S+app regressions were not significantly different from zero. In addition, a significant S+ × trial interaction was apparent from an ANCOVA in the control group ($F(1,76)$ = 6.83, P = 0.01) and to a lesser extent in the reconsolidation group ($F(1,76)$ = 2.43, P = 0.11). ANCOVAs directly comparing S+app and S+av regression lines across the groups revealed no significant group differences in S+ specific percentage 'Go' responses over trials. **(B)** Differential percentage 'Go' responses to the stimuli (S+app - S+av) over trials, in each group. An ANCOVA test for differences in these regressions revealed no main effect or interaction, as was the case in the the S+ specific group comparisons. **(C)** Percentage 'Go' responses over trials in each round separately (all subjects). In both rounds, the slope of the S+app regression was not significantly different from zero but the slope of the S+av regression was significantly negative (round 1, β = -0.2, P < 0.05; round 2, β = -0.42, P < 0.0001). Additionally, an ANCOVA revealed that there was a main effect of S+ in round 2 ($F(1,76)$ = 9.14, P < 0.005) and a S+ × trial interaction in both rounds (round 1, $F(1,76)$ = 3.32, P = 0.07; round 2, $F(1,76)$ = 7.37, P < 0.01).

Test trials

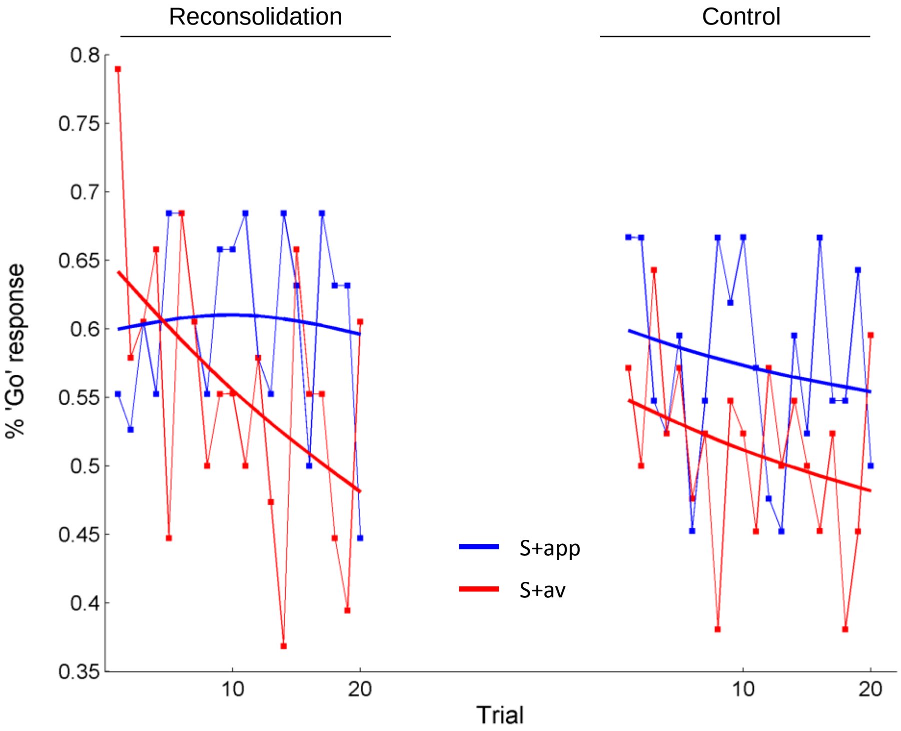

Supplementary Figure 2. Test session trial-by-trial percentage 'Go' responses to the S+app and S+av, by group (averaged over rounds).

ANCOVAS revealed main effects of S+ (S+app > S+av; control $F(1,36) = 7.84$, $P < 0.01$, reconsolidation $F(1,36) = 3.7$, $P = 0.06$) and a trend interaction in the reconsolidation group ($F = 3.33$, $P = 0.076$). The only regression line with a slope significantly different to zero was for the S+av in the reconsolidation group ($\beta = -0.84$, $P < 0.05$). Note that the reconsolidation group results were skewed by an outlier for the S+av in the first trial. When this was removed the regression for the S+av was also not significantly different from zero and results of the ANCOVA were more similar to the control (main effect of S+app > S+av, $F(1,34) = 6.5$, $P < 0.025$; no significant interaction). Direct group comparisons in this measure – by means of an ANCOVA of the differential percentage

'Go' responses – revealed no significant difference in performance (main effect or interaction) on test trials. ANCOVAs directly comparing the individual S+app and S+av lines across groups also revealed no differences (these group comparisons included the outlying trial mentioned above).

Day 2

Non-discriminatory learning (phase 2) trials

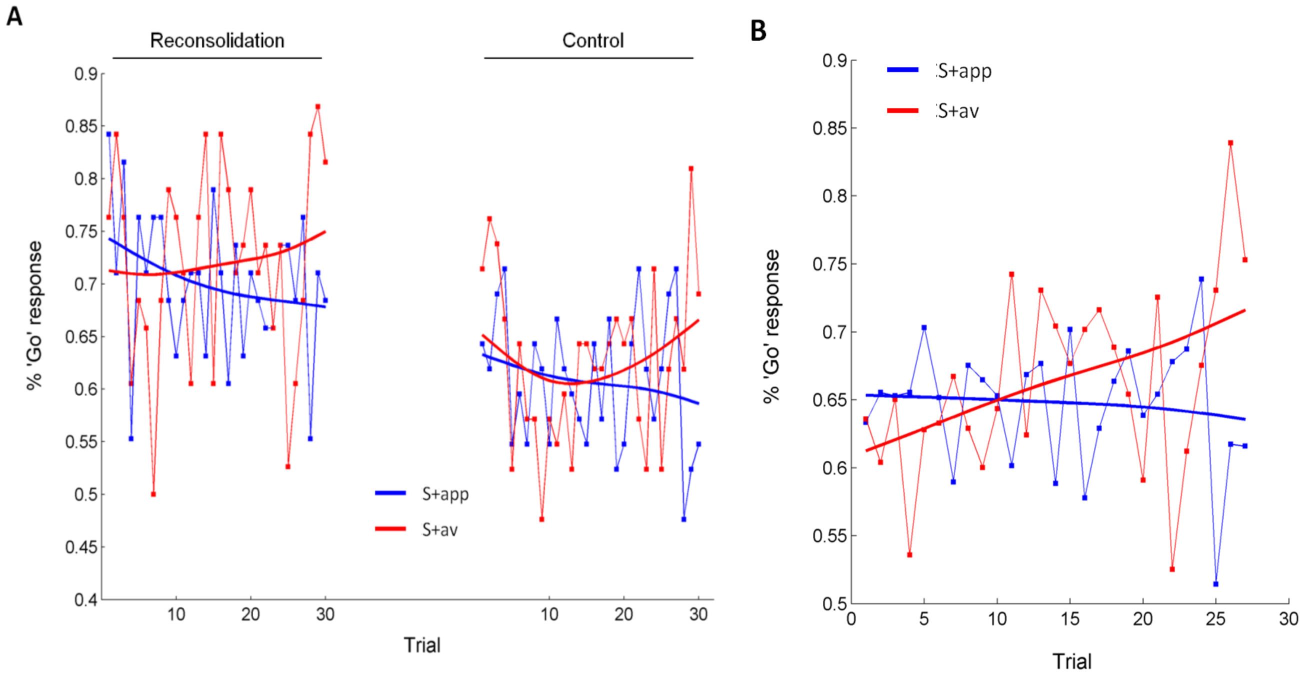

Supplementary Figure 3. Trial-by-trial percentage 'Go' responses to the S+app and S+av during phase 2 learning (scored according to contingencies on day 1 and averaged over rounds).

(A) The between groups ANCOVA revealed no significant differences in the regressions of the differential percentage 'Go' responses, nor were there any significant between group differences in the individual S+app and S+av regressions. **(B)** Percentage 'Go' responses of all subjects during phase 2 learning, excluding the first three trials (cf. Fig.

4). When excluding the first three trials on day 2 (trials where the percentage of 'Go' responses were very high compared to subsequent trials; Fig. 4), the regression slope of the S+av was significantly positive ($\beta = 0.4$, $P < 0.05$) and there was a significant S+app × S+av interaction in the ANCOVA ($F(1,50) = 5.28$, $P = 0.025$).

Day 3

Preferences in Reconsolidation group

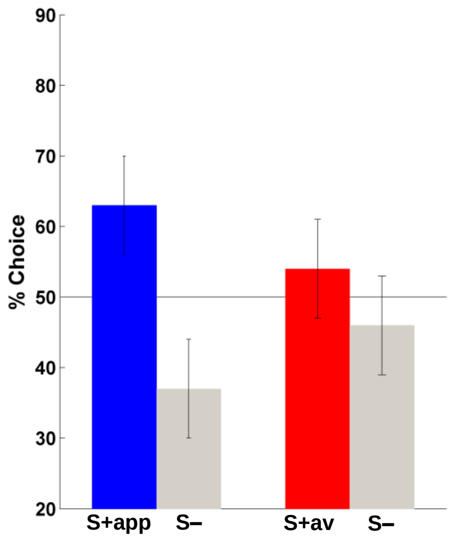

Supplementary Figure 4. Preferences in the Reconsolidation group for S+ vs. S- choices.

Both the Aversive and the Appetitive stimuli were preferred to the neutral cues. No statistics were performed on these individual choice categories in the reconsolidation group since the ANOVA showed that the distribution of preferences over all choices did not differ than what would be expected under the null hypothesis, that cues should be equally preferred over all choices (Results; Fig. 5).
